# The systemic effect of PEG-nGO-induced oxidative stress in vivo in a rodent model

**DOI:** 10.3762/bjnano.10.91

**Published:** 2019-04-18

**Authors:** Qura Tul Ain, Samina Hyder Haq, Abeer Alshammari, Moudhi Abdullah Al-Mutlaq, Muhammad Naeem Anjum

**Affiliations:** 1 Department of Physics, The Islamia University of Bahawalpur, Pakistan; 2 Department of Physics and Astronomy, King Saud University, Kingdom of Saudi Arabia; 3 Department of Biochemistry, King Saud University, Kingdom of Saudi Arabia

**Keywords:** nano-graphene oxide, nanomedicine, oxidative stress, PEGylation

## Abstract

Oxidative stress (OS) plays an important role in the pathology of certain human diseases. Scientists have developed great interest regarding the determination of oxidative stress caused after the administration of nano-graphene composites (PEG-nGO). Graphene oxide sheets (GOS) were synthesized via a modified Hummer's method and were characterized by X-ray diffraction (XRD), ultraviolet–visible spectroscopy (UV), and transmission electron microscopy (TEM). The method of Zhang was adopted for cracking of GOS. Then nano-graphene oxide was PEGylated with polyethylene glycol (PEG). PEGylation of nGO was confirmed by Fourier-transform infrared spectroscopy (FTIR), UV spectroscopy and TEM. The average size distribution of nGO and PEG-nGO was determined by using dynamic light scattering (DLS). Subsequently, an in vivo study measuring a marker for oxidative stress, namely lipid peroxides, as well as antioxidant agents, including catalase, superoxide dismutase, glutathione, and glutathione S-transferase was conducted. A comparison at different intervals of time after the administration of a dose (5 mg/kg) of PEG-nGO was carried out. An increase in free radicals and a decrease in free radical scavenging enzymes in organs were observed. Our results indicated that the treatment with PEG-nGO caused an increased OS to the organs in the first few hours of treatment. However, the liver completely recovered from the OS after 4 h. Brain, heart and kidneys showed an increased OS even after 4 h. In conclusion increased OS induced by PEG-nGO could be detrimental to brain, heart and kidneys.

## Introduction

The recent progress in nanoscience and nanotechnology that has facilitated the synthesis of advanced nanomaterials has led to the development of effective drug delivery systems [1–2]. Graphene, a single layer of carbon atoms with a hexagonal 2-dimensional crystal structure, its use in nanoscience and nanotechnology, is of great interest for scientists. The hydrophobic nature of graphene restricts its use for biomedical applications. Scientists have overcome this challenge through the oxidation of graphene by an improved Hummer’s method [3]. Graphene oxide (GO), due to its hydrophilic nature, can host a large number of biocompatible polymers, such as chitosan [4], polyethylene glycol (PEG) [5], poly(ε-caprolactone) (PCL) [6], hydroxypropyl-β-cyclodextrin (HPCD) [7], and poly(L-lactic acid) (PLLA) [8]. Biocompatible GO has many prospective uses in tissue engineering [9], drug delivery [10], cancer therapy [11–12], and treatment of bacterial infections [13–14]. Dinescu et al. designed a chitosan 3D scaffold and enhanced its bioactivity, mechanical properties, and pore formation with GO for optimal bone tissue engineering [15]. Zhang et al. improved the chemotherapy efficacy of anticancer drugs with polyethyleneimine (PEI)-grafted GO [16]. Liu et al. discussed the antibacterial activity of GO [17]. Moreover, GO-based sensors have been used for the detection of neonicotinoids [18], tyrosine [19], ascorbic acid, dopamine, uric acid [20], 4-nitrophenol [21], and glucose [22]. Among all biocompatible polymers, PEG has been extensively used as a GO cover. Feng et al. used PEG and PEI dual-functionalized GO for the photothermal enhancement of gene delivery [23]. Xiong et al. studied the synergistic effects of PEG-functionalized GO for chemo-photothermal therapy [24]. Tian et al. revealed that PEG-GO enhanced the uptake of chlorin e6 by cancer cells [25]. Shen et al. exploited the ability of PEGylated GO (PEG-GO) to deliver proteins into cells [26]. In addition, functionalized PEG-GO has been used as a nano-carrier of photosensitizers and synergistic anticancer agents [27].

PEG-GO has been widely used in vivo studies. Li et al. demonstrated that PEG coating reduced the retention of nGO in organs including lung, liver, and spleen and promoted its clearance from these organs [28]. Zhang et al. treated a tumour with a chemo-photothermal therapy based on PEG-GO [29]. The safety and tumour accumulation of PEG-GO has been reported by Miao and co-workers [30]. Functionalized PEG-nGO has been utilized as a potent radiotracer and drug-delivery agent in vivo using positron emission tomography (PET) imaging by Jang and co-workers [31]. Liu et al. [32–33] used PEG for the first time to functionalize GO, which can then be use as a vehicle for anticancer drugs such as doxorubicin. The combination of GO and drug led to a significant enhancement of the chemotherapy efficacy. The bio-distribution and toxicity of PEG-GO was examined by Yang and co-workers [5]. Their results clearly indicated that PEG-GO mainly accumulated in reticuloendothelial systems in liver and spleen after intravenous administration and can be cleared gradually by renal and fecal excretion. Furthermore, a number of in vitro studies indicated that treatment of various cell lines, such as 3T3 and Hela, greatly reduced the cellular viability [34]. Due to the contradictory study results, the applicability of PEG-GO for drug delivery in clinical use remains unclear.

Reactive oxygen species (ROS) have the potential to cause tissue destruction [35] and play an important role in the pathology of certain human diseases including atherosclerosis [36], rheumatoid arthritis [37], cancer [38], and neurodegenerative diseases [39]. Raised intracellular levels of ROS cause oxidative stress in cells [40]. Aerobic organisms alleviate the damaging effects of ROS with antioxidant systems comprising enzymatic and non-enzymatic antioxidants [41]. Our research aims to determine the oxidative stress induced through the administration of PEG-nGO and systematically evaluate its effect on organs for up to 4 h. The purpose of this study is also to assess the toxicity of PEG-nGO on different organs, which could help us to evaluate in detail the impact of these nanoparticles on different organs for its future use in medicine.

## Results

### X-ray diffraction

**Figure 1:**
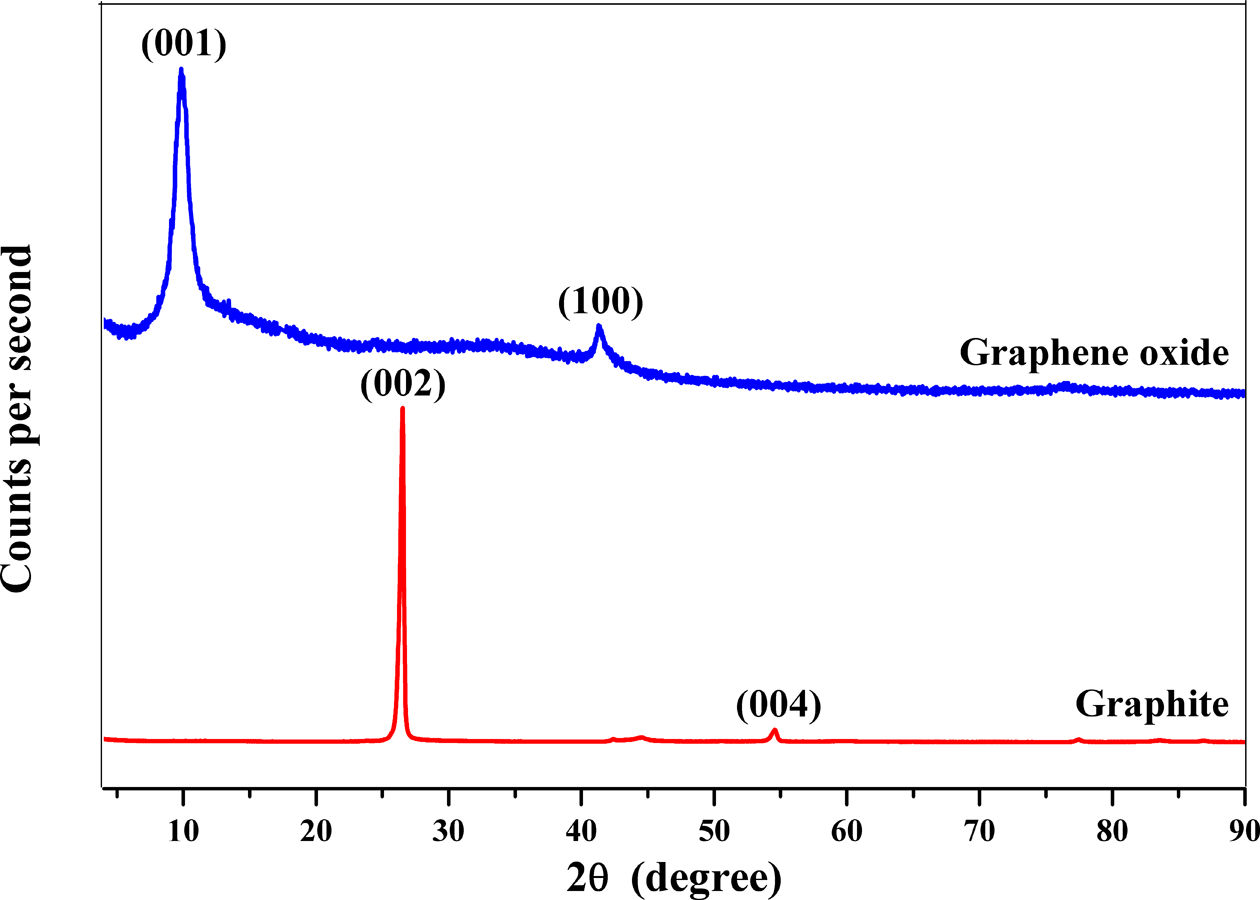
X-ray diffraction (XRD) spectra of pure graphite and synthesized graphene oxide. Deviation of XRD peak at 2θ = 26.5° to 9.9° confirmed the successful oxidation of graphite sheets.

X-ray diffraction (XRD) was carried out to identify the structure of the cellular units (*d*-spacing) used for the confirmation of a successful GO synthesis. A Bruker D8 ADVANCE diffractometer with Cu Kα radiation (λ = 1.54060 Å) was used. Figure 1 shows the XRD patterns. In the case of pure graphite, a strong XRD peak at 2θ = 26.5° and a slight peak at 2θ = 54.5° were observed specific to the (002) and (004) planes with *d*-spacing of 3.5 Å and 1.9 Å, respectively. The XRD pattern of graphene oxide shows a peak at 2θ = 9.9° (*d*-spacing of 8.9 Å), the (100) diffraction peak at 2θ = 42.0° according to a *d*-spacing of 2.13 Å, confirming the successful GO synthesis [42].

### Fourier-transform infrared spectroscopy

**Figure 2:**
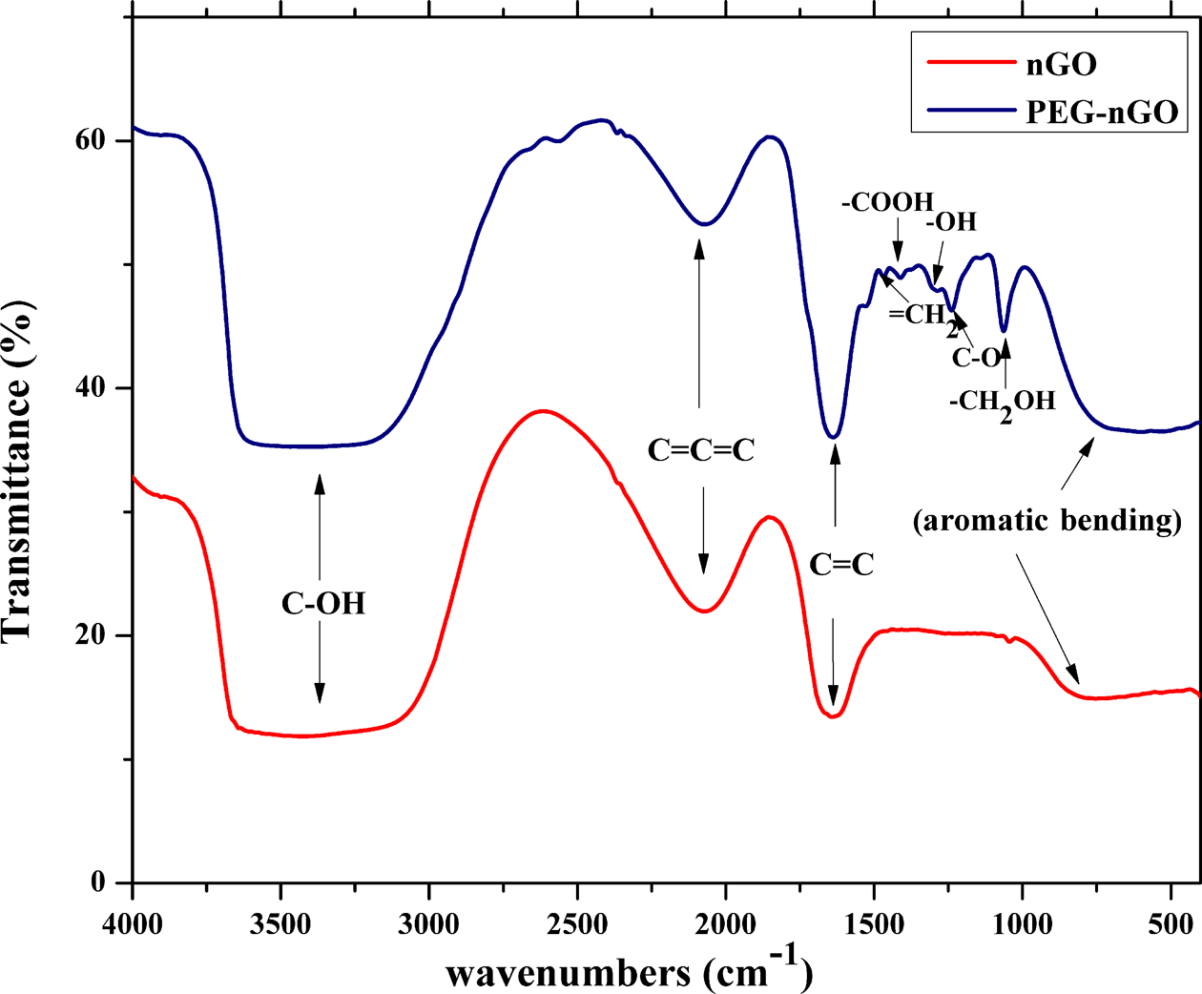
FTIR spectra of nGO and PEG-nGO. Additional peaks in the fingerprint region demonstrate the successful PEGylation of nGO.

The surface functional groups of nGO and PEG-nGO were investigated by Fourier-transform infrared spectroscopy (FTIR). The infrared spectra of nGO and PEG-nGO were processed using Origin Pro, and are shown in Figure 2.

In the case of nGO, a broad absorption band in the range of 3700–3000 cm^−1^ indicates the presence of free and intermolecular bonded hydroxy groups. The allene symmetry of nGO is recognized by an absorption band at 2000 cm^−1^. Another absorption band at 1650 cm^−1^ appears due to stretching of the cyclic alkene (C=C). The absorption band at 800 cm^−1^ appears due to bending of the aromatic structure of nGO. The infrared spectrum of PEG-nGO is similar to that of nGO with additional peaks in the fingerprint region, indicating an expansion of the vibrational stretching of hydroxy groups, a bending of the aromatic structure, and a reduction in the allene symmetry of nGO. The presence of the methylene groups (=CH_2_), carboxyl groups (–COOH), and phenol groups (–OH) in PEG-nGO is shown through the absorption peaks at 1465 cm^−1^, 1410 cm^−1^, and 1300 cm^−1^, respectively. The vibrational stretching at 1240 cm^−1^ and 1060 cm^−1^ is from aromatic esters and primary alcohols (–CH_2_OH), respectively. Moreover, an increase in the aromatic bending is observed [43].

### Ultraviolet–visible spectroscopy

**Figure 3:**
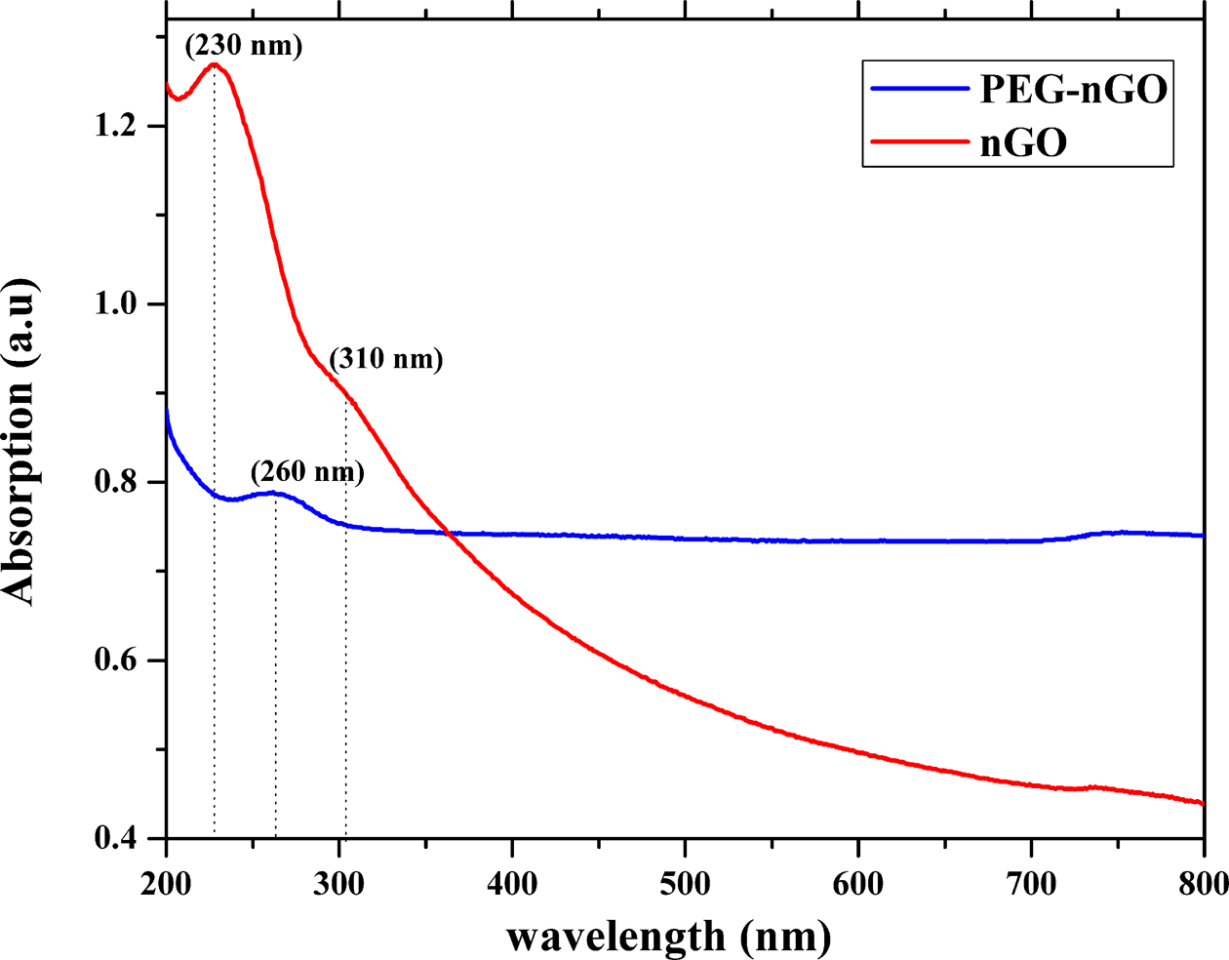
UV–vis spectra of nGO and PEG-nGO. The red-shift of the UV peak confirmed the nGO loading with PEG.

The UV–vis absorption spectra of nGO and PEG-nGO are shown in Figure 3. The absorption spectra of nGO shows an absorption at λ_max_ = 230 nm and a second shoulder peak at 310 nm. The sharp absorption peak at 230 nm is connected to the π–π* electronic transition in carbon–carbon bonds and the shoulder peak is related to the n–π* electronic transition of non-bonding electrons of peroxide and epoxide groups [42]. A red-shift of λ_max_ to 260 nm is observed for PEG-nGO [44].

### Transmission electron microscopy

**Figure 4:**
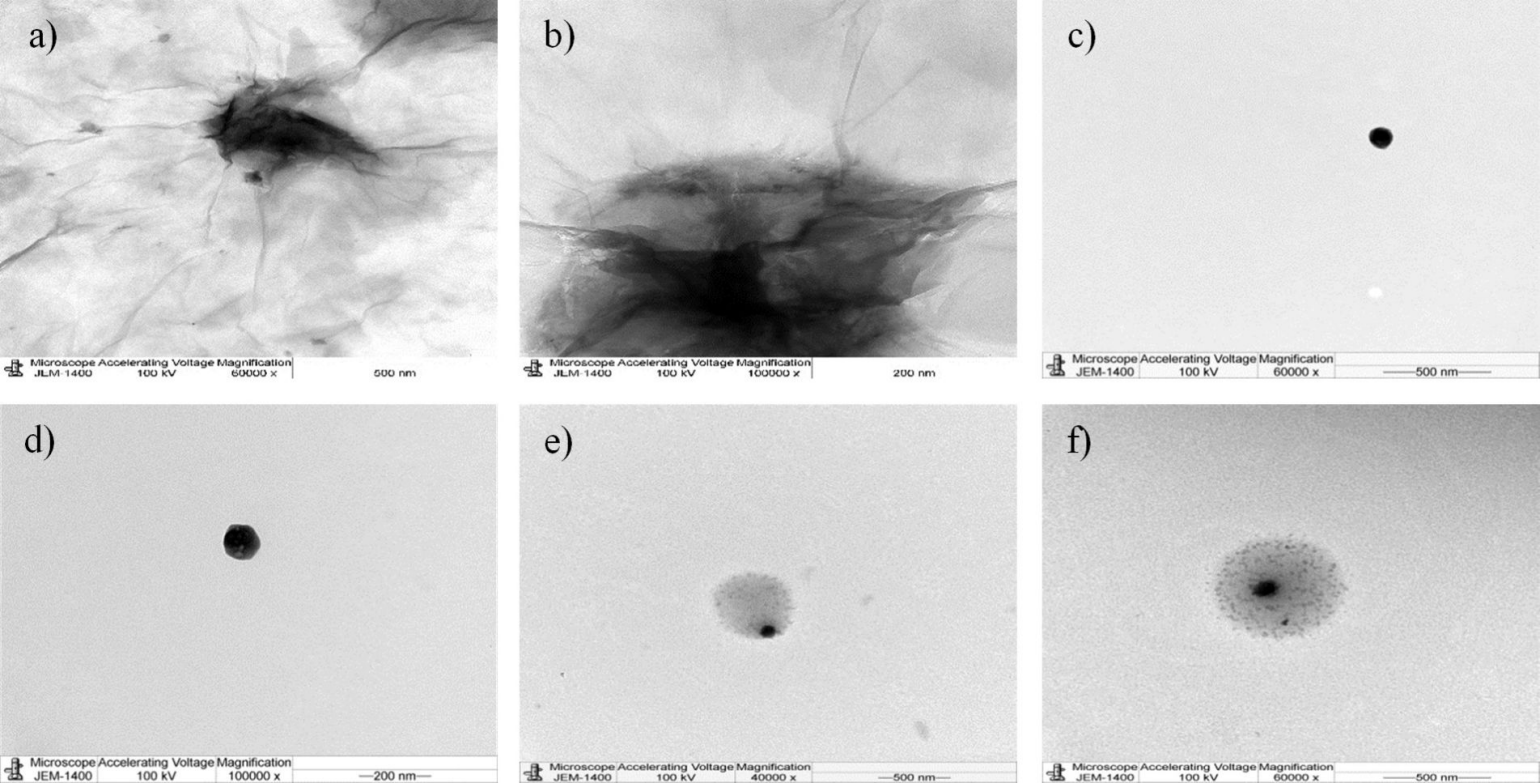
TEM images of (a,b) graphene oxide, (c,d) nano-graphene oxide, and (e,f) PEGylated nano-graphene oxide.

Transmission electron microscopy (TEM) was used to examine the morphology of GO, nGO, and PEG-nGO. A wrinkled sheet of GO, and round-shaped nGO, appeared under TEM (JEOL TEM-1400 Plus, operated at a voltage of 100 kV and a current of 54 μA). The different apparent transparencies of the GO sheet indicate the variable thickness of the GO sheet. The dark area shows thick wrinkled stacking of GO layers, while the transparent area reveals single-layer or few-layer GO [45]. Also, a thick covering of PEG is clearly seen around nGO (Figure 4).

### Dynamic light scattering

**Figure 5:**
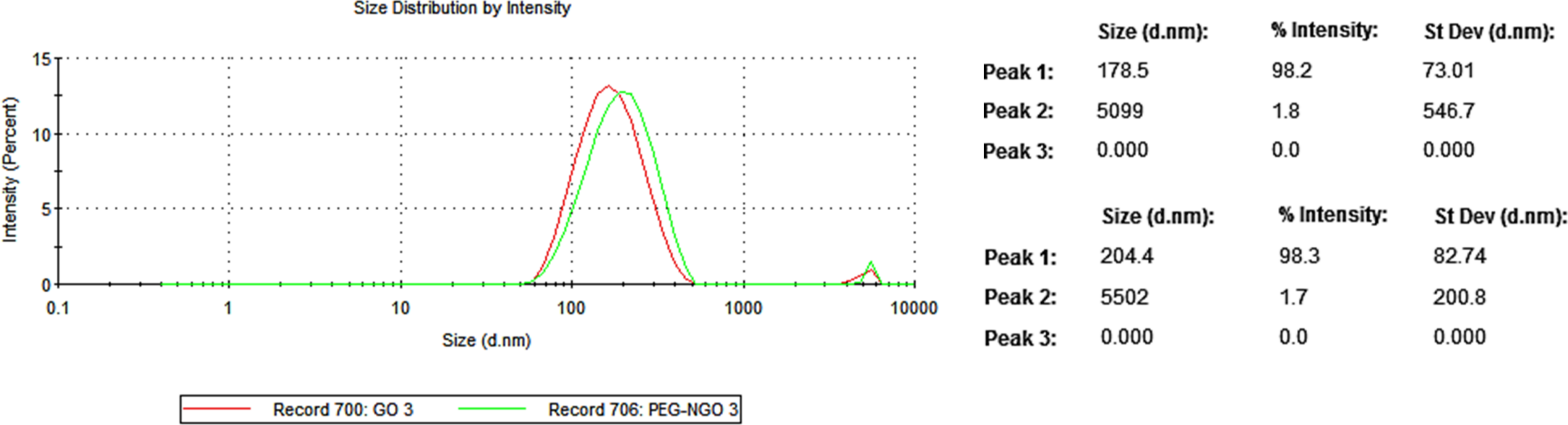
Particle size distribution curves for nGO and PEG-nGO.

The average particle size distributions of nGO and PEG-nGO were determined by dynamic light scattering (Malvern Panalytical ZS90, Figure 5). The intensive peaks were recorded at 178.5 ± 73.01 nm and 204 ± 82.74 nm, indicating the average size distribution of nGO and PEG-nGO, respectively.

### In vivo assays

**Figure 6:**
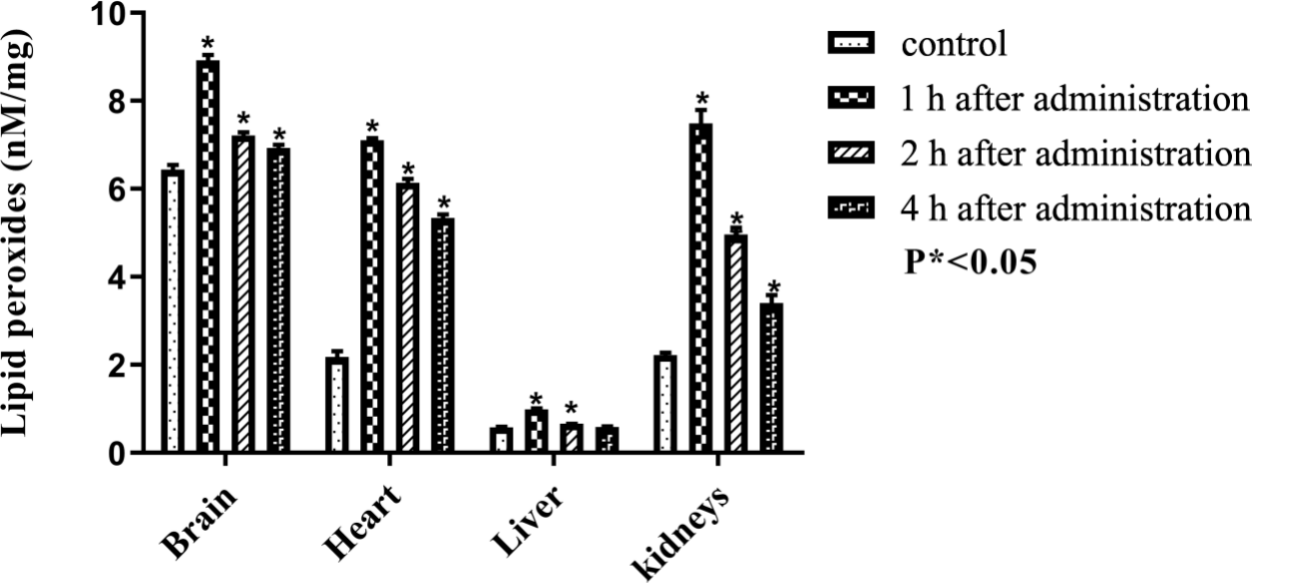
Levels of lipid peroxides after intraperitoneal administration of PEG-nGO; error bars: one standard deviation.

**Figure 7:**
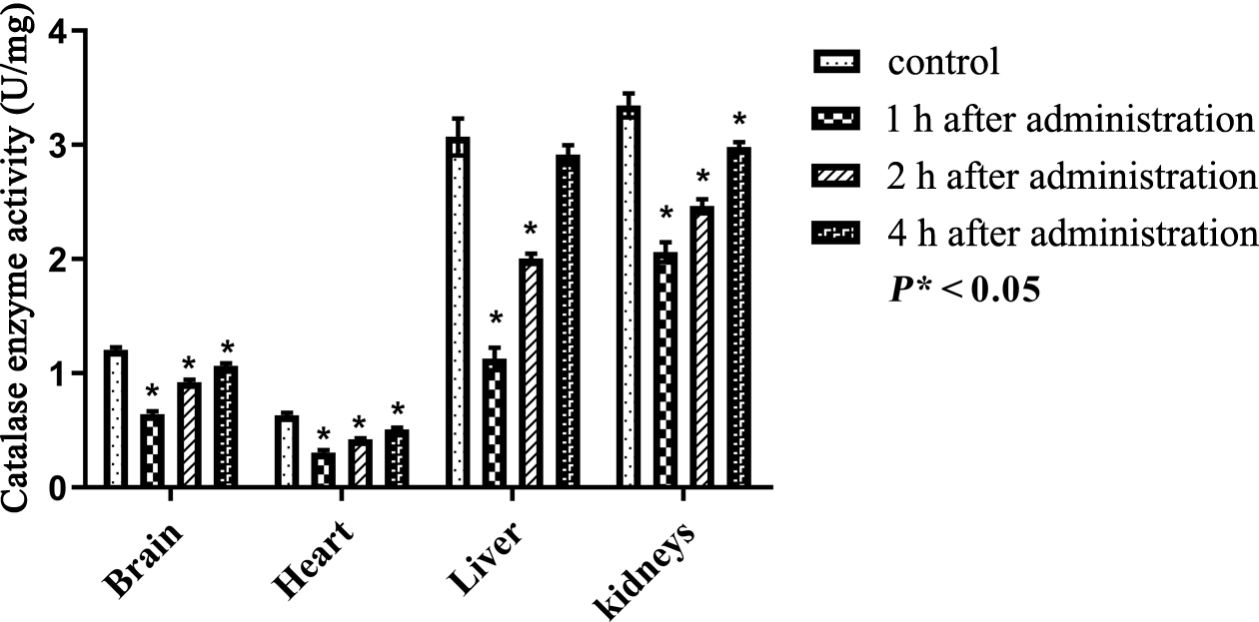
Catalase antioxidant enzyme activity after intraperitoneal administration of PEG-nGO, error bars: one standard deviation.

**Figure 8:**
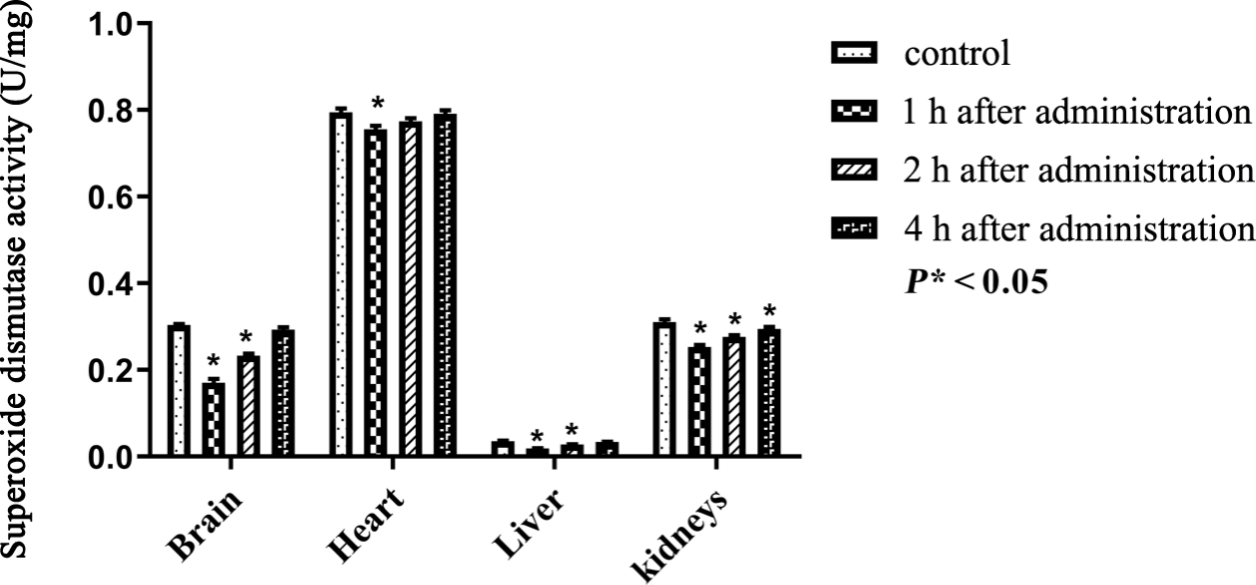
Superoxide dismutase activity after intraperitoneal administration of PEG-nGO, error bars: one standard deviation.

**Figure 9:**
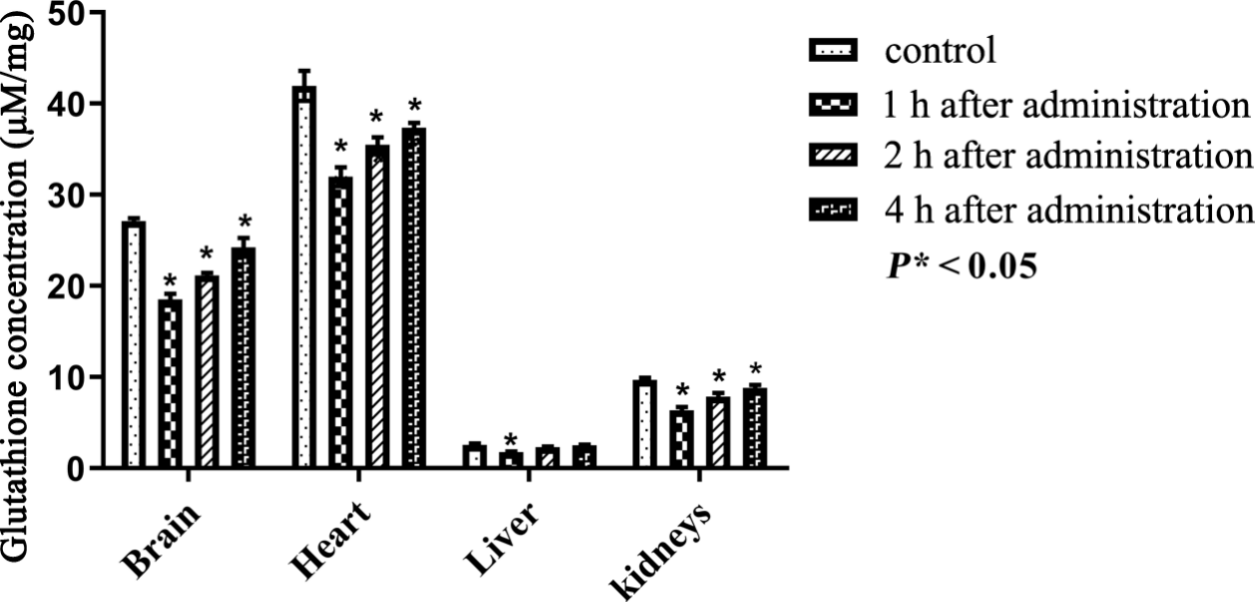
Concentration of reduced glutathione after intraperitoneal administration of PEG-nGO, error bars: one standard deviation.

**Figure 10:**
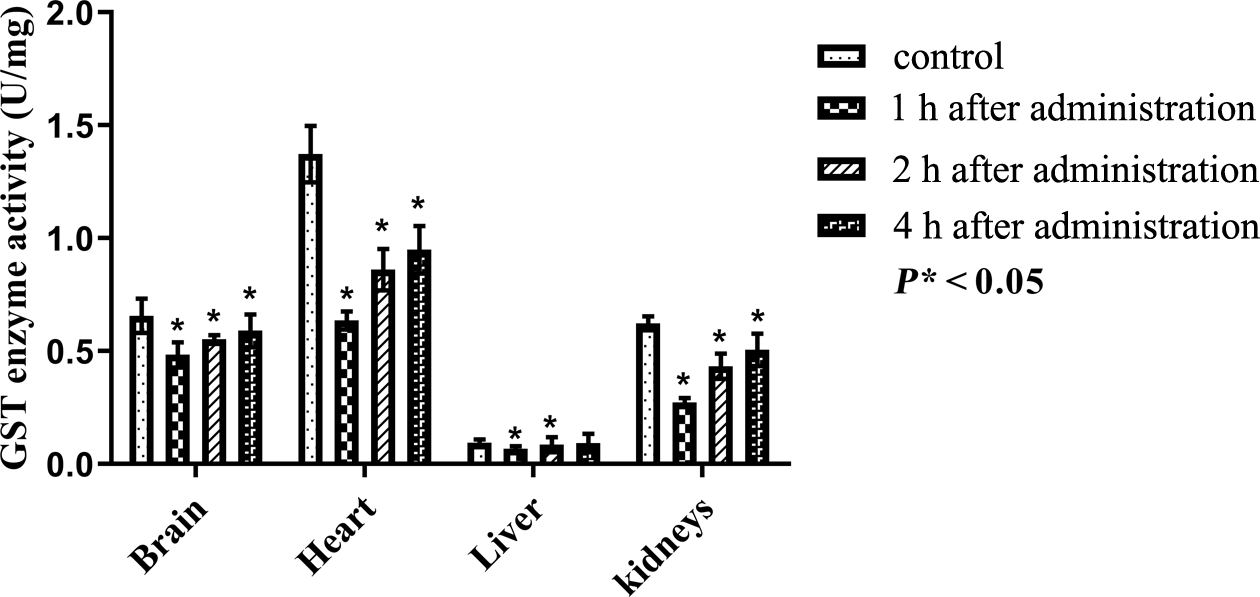
Activity of glutathione S-transferase enzyme activity after intraperitoneal administration of PEG-nGO, error bars: one standard deviation.

The number of free radicals produced by a dose of PEG-nGO was estimated by the comparative levels of lipid peroxides present in the control and treated groups. The level of lipid peroxides and free radical scavengers are shown in Tables 1–5 and Figures 6–10. One hour after the intraperitoneal administration of PEG-nGO (5 mg/kg), the concentrations of lipid peroxidation marker malondialdehyde (MDA), reduced free radical scavenging enzymes (CAT, SOD, GST), and the tripeptide scavenger glutathione (GSH) was significant (P* < 0.05) in the brain, heart, liver, and kidneys of the treated groups. The MDA concentration level showed an increase to 140%, 330%, 170%, and 340% in brain, heart, liver and kidneys, respectively. Tissues of heart and kidneys are greatly influenced by PEG-nGO, as the detected level of MDA was more than two times than that of the control group. A reduction of the increased MDA level with time was monitored in all organs. However, 4 h after PEG-nGO administration, the level of MDA was still significantly increased (P* < 0.05) in brain, heart, and kidneys.

**Table 1:** Concentration (nM/mg) of lipid peroxides measured after administration of PEGylated nano-graphene oxide in different organs after different periods of time (mean ± standard deviation).

organ	control	after 1 h	P*	after 2 h	P*	after 4 h	P*
	(*n* = 6)	(*n* = 6)		(*n* = 6)		(*n* = 6)	

brain	6.44 ± 0.04	8.92 ± 0.05	<0.05	7.21 ± 0.03	<0.05	6.92 ± 0.02	<0.05
heart	2.18 ± 0.05	7.11 ± 0.06	<0.05	6.18 ± 0.06	<0.05	5.34 ± 0.07	<0.05
liver	0.58 ± 0.01	0.99 ± 0.01	<0.05	0.66 ± 0.01	<0.05	0.58 ± 0.01	—
kidneys	2.22 ± 0.03	7.64 ± 0.09	<0.05	4.96 ± 0.06	<0.05	3.4 ± 0.07	<0.05

Catalase (CAT) is an important free radical scavenging enzyme that converts hydrogen peroxide (H_2_O_2_) into water and oxygen, thus preventing cell damage. A reduction of the acitivity of CAT after PEG-nGO administration was observed as shown in Table 2.

**Table 2:** Catalase activity normalised to the wet-tissue mass (U/mg) after administration of PEGylated nano-graphene oxide in different organs after different periods of time (mean ± standard deviation).

organ	control	after 1 h	P*	after 2 h	P*	after 4 h	P*
	(*n* = 6)	(*n* = 6)		(*n* = 6)		(*n* = 6)	

brain	1.2 ± 0.01	0.64 ± 0.01	<0.05	0.92 ± 0.01	<0.05	1.07 ± 0.01	<0.05
heart	0.63 ± 0.01	0.3 ± 0.01	<0.05	0.42 ± 0.003	<0.05	0.51 ± 0.01	<0.05
liver	3.07 ± 0.07	1.13 ± 0.04	<0.05	2.01 ± 0.02	<0.05	2.91 ± 0.04	—
kidneys	3.35 ± 0.04	2.06 ± 0.04	<0.05	2.46 ± 0.02	<0.05	2.98 ± 0.02	<0.05

One hour after PEG-nGO administration, the decrease in CAT enzyme activity was significant (P* < 0.05) in all organs (Figure 7). The decrease in the CAT enzyme is related to the increased level of MDA. Four hours after administration the CAT enzyme activity was still significantly decreased in all organs except the liver.

Superoxide dismutase (SOD) is another free radical scavenging enzyme. Three kinds of SOD exist in all organs of mammals. SOD1, SOD2, and SOD3 are present in the cytoplasm, mitochondria, and extracellular, respectively. SOD converts the superoxide (O_2_^−^) produced due to the xenobiotic into oxygen and hydrogen peroxide. The amount of SOD present in the brain, heart, liver, and kidneys of the control and treated groups is shown in Table 3.

**Table 3:** Activity of superoxide dismutase normalised to the wet-tissue mass (U/mg) after administration of PEGylated nano-graphene oxide in different organs after different periods of time (mean ± standard deviation).

organ	control	after 1 h	P*	after 2 h	P*	after 4 h	P*
	(*n* = 6)	(*n* = 6)		(*n* = 6)		(*n* = 6)	

brain	0.304 ± 0.001	0.171 ± 0.004	<0.05	0.233 ± 0.002	<0.05	0.293 ± 0.002	—
heart	0.795 ± 0.004	0.756 ± 0.003	<0.05	0.774 ± 0.003	—	0.791 ± 0.003	—
liver	0.035 ± 0.0006	0.019 ± 0.0003	<0.05	0.028 ± 0.0002	<0.05	0.034 ± 0.0004	—
kidneys	0.311 ± 0.003	0.253 ± 0.002	<0.05	0.276 ± 0.002	<0.05	0.295 ± 0.002	<0.05

The activity response of SOD was similar to that of CAT in all organs except the heart (Figure 8). In the heart, the initial activity of SOD remained, indicating a full recovery from the induced OS. After 4 h, the observed SOD activity levels were no more significantly (P* < 0.05) increased in brain and liver, indicating a recovery from the induced OS.

Glutathione is a tripeptide and, in its reduced form, acts as an active scavenger of free radicals produced as a result of lipid peroxidation. The concentration levels of the reduced glutathione in organs before and after the administration of PEG-nGO are indicated in Table 4. The observed significant decrease (P* < 0.05) and subsequent increase of the concentration of glutathione is correlated the state of oxidative stress in the organs (Figure 9).

**Table 4:** Concentration (μM/mg) of reduced glutathione in different organs after administration of PEGylated nano-graphene oxide in different organs after different periods of time (mean ± standard deviation).

organ	control	after 1 h	P*	after 2 h	P*	after 4 h	P*
	(*n* = 6)	(*n* = 6)		(*n* = 6)		(n = 6)	

brain	27.09 ± 0.06	18.50 ± 0.17	<0.05	21.16 ± 0.03	<0.05	24.24 ± 0.43	<0.05
heart	41.95 ± 1.09	31.99 ± 0.44	<0.05	35.97 ± 0.29	<0.05	37.34 ± 0.12	<0.05
liver	2.57 ± 0.01	1.76 ± 0.003	<0.05	2.29 ± 0.005	—	2.54 ± 0.002	—
kidneys	9.7 ± 0.02	6.36 ± 0.05	<0.05	7.86 ± 0.06	<0.05	8.79 ± 0.05	<0.05

The activity of glutathione S-transferase (GST) plays a central role in preventing damage to tissues. The GST enzyme activity is shown in Table 5 and Figure 10. It was found that PEG-nGO reduced GST enzyme activity in the tissues of all organs.

**Table 5:** Activity of GST normalised to the wet-tissue mass (U/mg) after administration of PEGylated nano-graphene oxide in different organs after different periods of time (mean ± standard deviation).

organ	control	after 1 h	P*	after 2 h	P*	after 4 h	P*
	(*n* = 6)	(*n* = 6)		(*n* = 6)		(*n* = 6)	

brain	0.656 ± 0.02	0.483 ± 0.02	<0.05	0.552 ± 0.01	<0.05	0.591 ± 0.03	<0.05
heart	1.373 ± 0.05	0.636 ± 0.02	<0.05	0.861 ± 0.02	<0.05	0.949 ± 0.04	<0.05
liver	0.094 ± 0.006	0.067 ± 0.002	<0.05	0.085 ± 0.002	<0.05	0.093 ± 0.003	—
kidneys	0.623 ± 0.01	0.272 ± 0.01	<0.05	0.433 ± 0.02	<0.05	0.506 ± 0.02	<0.05

## Discussion

In recent years various forms of graphene oxide, especially its conjugated form with PEG has gained considerable attention and interest due to its unique physiochemical properties [46]. The functionalization of GO with PEG has been reported to improve the stability in aqueous solution and the biocompatibility, making it an ideal drug-delivery vehicle [47–48]. Rapid progress has been made in the synthesis of graphene oxide nanoparticles and their use in medicine, so there is an urgent need to look at the safety and efficacy of these particles in vivo. It has been observed that engineered nanomaterials, e.g., from metals or metal oxides, have the potential to induce toxicity by generating free radicals [49]. These free radicals can attack the surrounding biological molecules such as proteins, lipids and even DNA, which could result in a loss or damage of their biological function. In the body of mammals, there are antioxidants to counter these free radicals and protect tissues from damaging effects. These antioxidants include both enzymes such as GST, catalase and SOD, and non-enzymatic antioxidants such as reduced glutathione, vitamin E and many polyphenolic compounds.

This study aimed to examine the oxidative stress, which is a major cause of organ damage after the administration of PEGylated nano-graphene oxide (PEG-nGO), and examined the recovery of the organ from the stress after specific intervals of time. For this purpose, we oxidized sheets of graphite with an improved Hummer's method [3]. Afterward, GO sheets were cracked into nGO by a previously adopted method by Zhang and co-workers [29]. Biocompatibility of nGO was achieved by coating with polyethylene glycol (PEG). Then, a single dose (5 mg/kg) of synthesized PEG-nGO was administered to groups of groups of six mice. The dosage was found to be optimum in previous studies [28–29]. The animals were dissected after specific intervals of time. Lipid peroxide marker and reduced GSH as well as enzymes, including catalase, superoxide dismutase and glutathione S-transferase, were monitored in brain, heart, liver, and kidneys.

The interactions of graphene nanoparticles with the membrane integrity has been studied and found that treatment with GO can extract phospholipid and cholesterol from the plasma membrane of human alveolar epithelial A549 cells, producing surface pores [50–51]. This effect greatly reduced the cell viability and results in cellular damage and apoptosis, and long-term exposure could cause organ damage.

Our data suggest that PEG-nGO induced a high oxidative stress (OS) to the organs in the first hour of treatment, as assessed by the increased concentration levels of malondialdehyde (MDA), an end product of the peroxidation of polyunsaturated fatty acids (PUFA). As cell membranes contain great amounts of PUFA, high levels of MDA indicate damaged cell membranes. MDA is commonly used as a marker for oxidative stress and the antioxidant status of the respective organ. It was also show that tissues of heart and kidneys are greatly influenced by PEG-nGO. A decreased amount of the antioxidant enzymes CAT, SOD, and GST was also revealed. The increased amount of MDA, and the reduced concentration levels of antioxidant enzymes directly indicate the tissue damage after the uptake of PEG-nGO. Our findings demonstrate the slow recovery of organs. After 4 h of administration, the organs were still found to be under oxidative stress with the exception of the liver. The reduced retention of PEG-nGO in the liver is in accordance with results previously described by Li and co-workers [28].

## Conclusion

In the present study, our aim was to examine the oxidative stress in organ tissues after a single-dose administration (5 mg/kg) of biocompatible nano-graphene oxide. The oxidative stress caused by oxidants such as lipid peroxide, and the activity of antioxidants, including catalase, superoxide dismutase, glutathione, and glutathione S-transferase, was monitored. The increased concentration of MDA accompanied by reduced activity levels of antioxidant enzymes directly indicated that all organs were in oxidative stress after the intraperitoneal administration of PEG-nGO. These studies further reiterated the cytotoxicity of graphite oxide in vivo. Further safety evaluation and research must be undertaken in order to establish the use of these biocompatible polymer nanoparticles to be used in human tissues for clinical applications.

## Experimental

### Materials

All materials were purchased from Sigma-Aldrich, St Louis, USA and used without further purification. The chemicals used for GO synthesis are graphite powder, potassium permanganate, phosphoric acid (85%), sulfuric acid (98%), and hydrogen peroxide (30%). Polyethylene glycol (PEG) was used for PEGylation. Thiobarbituric acid (TBA, 0.6%), and trichloroacetic acid (TCA, 20%) were used to estimate the malondialdehyde (MDA) level, sodium phosphate buffer, and hydrogen peroxide were used to monitor the catalase activity, phenazine methosulphate, nitro blue tetrazolium, sodium pyrophosphate buffer, nicotinamide adenine dinucleotide (NADH), and glacial acetic acid were used to detect superoxide dismutase (SOD) enzyme level, Ellman’s reagent [5,5′-dithiobis(2-nitrobenzoic acid), DTNB], and TCA were used to examine the reduced glutathione level, and CDNB (1-chloro-2,4-dinitrobenzene), and glutathione were used to measure GST activity.

### Graphene oxide synthesis

Highly oxidized sheets of graphene oxide (GO) were synthesized by an improved Hummer’s method [3]. Briefly, phosphoric acid (H_3_PO_4_) was added to sulphuric acid (H_2_SO_4_) in a 1:9 ratio. Then, 1.5 g of pure graphite powder was added into the solvent under stirring. After 5 min, 9 g of potassium permanganate (KMnO_4_) was slowly added to the solution. The solution was stirred for 24 h. After this, the oxidation process was stopped by the drop-wise addition of 10 mL of hydrogen peroxide (H_2_O_2_). The reaction was completely terminated by the addition of 200 mL of doubly distilled water. The synthesized GO was washed with distilled water several times and was dried at 80 °C overnight.

### PEGylation of graphene oxide

For PEGylation, the sheets of GO were cracked into nano-graphene oxide (nGO) by an ultrasonic probe at 200 W for 3 h [29]. Then, the nGO solution (2 mg/mL) was neutralized by repeated rinsing and was purified through filtration using a Millex^®^ syringe filter (0.45 μm). Finally, 400 mg of polyethylene glycol (PEG) was added to the solution which was stirred for 6 h. The PEG-nGO solution was centrifuged at 1000*g* for 10 min and was washed with distilled water several times and dried at 55 °C for 24 h.

### Characterization techniques

X-ray diffractometer (Bruker D8 ADVANCE) was used to confirm the GO synthesis from graphite powder. The presence of functional groups was analysed by using Fourier-transform infrared spectrometry (PerkinElmer Inc. Spectrum BX). Ultraviolet–visible absorption spectroscopy (Biochrom Libra S60PC) was used to identify the PEGylation of nGO. Transmission electron microscopy (JEOL JEM-1400 Plus) was used to examine the morphology of GO, nGO, and PEG-nGO. The average particle size distribution of nGO and PEG-nGO were analyzed by dynamic light scattering (Malvern Panalytical ZS90).

### In vivo treatment

Female albino mice were kept at the facility of King Saud University Research Center under the guidelines provided by the Experimental Animal Laboratory and approved by the Animal Care and Use Committee [52]. The mice were fed with standard mice chow and water ad libitum for two weeks at 23 ± 5 °C. The mice, with a weight of 23 ± 1 g, were divided into four groups of six mice. The administrated dose of PEG-nGO was estimated from previous studies [28–29]. Three groups were administered PEG-nGO (1 mL, 5 mg/kg) saline suspension while one group was administered saline (1 mL) only through an intraperitoneal (i.p.) injection. All groups were administrated once. The group that was administered saline only was treated as control group. To study the oxidative stress of PEG-nGO, the mice were anesthetized and were dissected after specific intervals of time (1 h, 2 h, and 4 h). After dissection, organs including the brain, heart, liver, and kidneys were carefully removed with the help of a sterilized scalpel and iris scissors. All organs were washed with saline and were homogenized with a total volume of 10 mL using sterilized phosphate-buffered saline (pH 7.4). All used protocols were approved by the Animal Ethics Committee of King Saud University, in accordance with the international guiding principles for biomedical research.

### In vivo assays

#### Lipid peroxidation

The amount of lipid peroxides (LOPs) in the collected organs of mice was estimated by TBA assay as described by Dubovskiy and co-workers [53]. Briefly, 1 mL of 0.6% thiobarbituric acid (TBA) was mixed with 0.25 mL of 20% trichloroacetic acid (TCA) in a glass tube covered by aluminum foil. Then, 0.5 mL of an enzyme (diluted tissue homogenate) was added to the mixture and kept in a water bath at 100 °C for 30 min. Afterward, the mixture was allowed to cool at room temperature and was centrifuged at 1000*g* for 10 min. The absorbance was measured at 535 nm using a UV–vis spectrometer. The level of LOPs was calculated as follows:


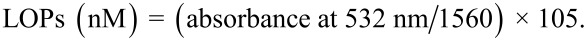


The calculated amounts of LOPs were normalised to the tissue mass. The total levels of LOPs are expressed as nM/mg.

#### Catalase activity

Catalase (CAT) activity was estimated in the whole homogenate according to the Aebi protocol [54]. Concisely, 0.5 mL of diluted enzyme (homogenized tissue) was added into 1.5 mL of (0.4 M) sodium phosphate buffer of pH 7.2. Then, 1 mL of hydrogen peroxide was added to the mixture and enzyme activity was measured at 240 nm for 2 min. Finally, CAT volume activity was calculated as follows:


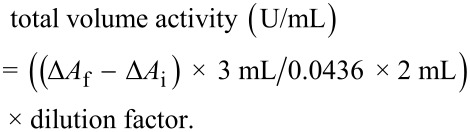


The extinction coefficient of hydrogen peroxide is 0.0436. The activity was normalised to the wet-tissue mass and is expressed as U/mg.

#### Superoxide dismutase

Superoxide dismutase (SOD) activity was measured by a slight modification of the method reported by Kakkar and co-workers [55]. Typically, 0.1 mL of phenazine methosulphate (186 µM), 0.3 mL of nitro blue tetrazolium (300 µM), 0.1 mL of enzyme (tissue homogenate), and 1 mL of distilled water were added to 1.2 mL of sodium pyrophosphate buffer (0.052 M, pH 8.3). Finally, 0.2 mL of NADH was added to the mixture. Then, the whole mixture was incubated at room temperature for 90 s. The reaction was stopped by the addition of 1 mL of glacial acetic acid. Then, the absorption was measured at 560 nm. SOD was calculated by the NBT method:


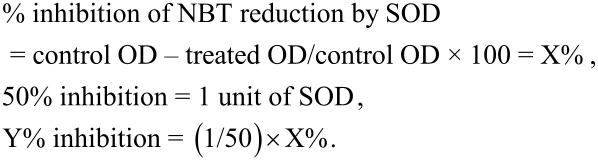


The SOD activity normalised to the wet-tissue mass and is expressed as U/mg.

#### Reduced glutathione assay

Ellman’s reagent [5,5′-dithiobis(2-nitrobenzoic acid)], as described by Moron and co-workers, was used to estimate the glutathione level [56]. Briefly, 1 mL of homogenized tissue was deproteinized by an equal volume of 20% TCA. The total mixture was centrifuged at 1000*g* for 10 min at 4 °C. The supernatant was mixed with a 0.04% DNTB buffer in a ratio of 1:9. Then, the absorbance was measured at 412 nm using a UV–vis spectrometer. The GSH level was determined using a calibration curve and was expressed in μM/mg.

#### Glutathione S-transferase

Glutathione S-transferase (GST) activity was calculated by a method described by Boyland and Chasseaud [57]. 10 µL of each, freshly prepared 1-chloro-2,4-dinitrobenzene and glutathione were added to 980 µL of PBS (pH 6.5). Then, 100 µL of the solution was replaced by tissue homogenate. Enzyme activity was measured at 340 nm until the reaction became linear. GST activity was calculated as follows:


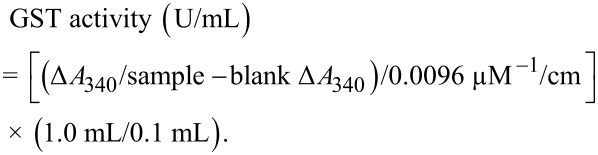


0.0096 µM^−1^/cm is the molar extinction of CDNB and 0.1 mL is the sample volume in the total volume of 1 mL.

### Statistical analysis

Data are presented as mean ± one standard deviation. Comparisons between the controlled and treated groups were analysed by one-way ANOVA using Microsoft Excel, and the values (P* < 0.05) were considered statistically significant.
